# Identification and Validation of Reference Genes for qRT-PCR Analysis of Petal-Color-Related Genes in *Rosa praelucens*

**DOI:** 10.3390/genes15030277

**Published:** 2024-02-23

**Authors:** Hongying Jian, Huichun Wang, Xianqin Qiu, Huijun Yan, Lulin Ma

**Affiliations:** Flower Research Institute, Yunnan Academy of Agricultural Sciences, Kunming 650205, China; ynwildflower@aliyun.com (H.J.); 17812085625@163.com (H.W.); xianqin711@hotmail.com (X.Q.); hjyan8203@126.com (H.Y.)

**Keywords:** *Rosa praelucens* Byhouwer, petal color variation, qRT-PCR, reference gene, petal-color-related genes

## Abstract

The flower’s color is regarded as one of the most outstanding features of the rose. *Rosa praelucens* Byhouwer, an endemic and critically endangered decaploid wild rose species, is abundant in phenotypic diversity, especially in flower color variation, from white to different degrees of pink. The mechanism underlying this variation, e.g., the level of petal-color-related genes, is worth probing. Seven candidate reference genes for qRT-PCR analysis, including tubulin α chain (*TUBA*), glyceraldehyde-3-phosphate dehydrogenase (*GAPDH*), histone H2B (*Histone2A*), eukaryotic translation elongation factor 1-α (*EEF1A*), 60S ribosomal protein (*RPL37*), eukaryotic translation initiation factor 1-α (*EIF1A*), and aquaporins (*AQP*), were detected from the transcriptome datasets of full blooming flowers of white-petaled and pink-petaled individuals, and their expression stabilities were evaluated through qRT-PCR analysis. According to stability rankings analysis, *EEF1A* showed the highest stability and could be chosen as the most suitable reference gene. Moreover, the reliability of *EEF1A* was demonstrated via qRT-PCR analysis of six petal-color-related target genes, the expression patterns of which, through *EEF1A* normalization, were found to be consistent with the findings of transcriptome analysis. The result provides an optimal reference gene for exploring the expression level of petal-color-related genes in *R. praelucens*, which will accelerate the dissection of petal-color-variation mechanisms in *R. praelucens*.

## 1. Introduction

*Rosa praelucens* Byhouwer refers to critically endangered wild rose species endemic to Shangri-La county in northwestern Yunnan, China [1,2]. As a well-known alpine ornamental plant [3] and an important rose germplasm resource with cold tolerance [4] and aphid resistance [5], *R. praelucens* has drawn considerable attention [6,7,8,9,10,11,12] since it was found to be the only extant natural decaploid species (2n = 10x = 70) with the highest number of chromosomes in the genus [13]. *R. praelucens* is abundant in phenotypic variation, among which flower color variation is most conspicuous [6,14]. In addition to the common color fading with the opening process of a single flower, the colors of full-bloom flowers from different individuals within or among populations show significant differences. Most individuals have flowers that are in different degrees of pink, while some individuals have white flowers.

Flower color has a vital impact on the attractiveness of rose flowers for insects, and has an important aesthetic value for humans. Inherited from different genetic background species, most flower colors, except blue, are naturally present in roses [15], mostly identified by the constituent profile of the chemicals, e.g., flavonoids and anthocyanins. Limited flower colors can be obtained within a species because of genetic constraints [16]. In roses, yellow and orange colors are caused by carotenoid pigment accumulation, while red colors are due to anthocyanin accumulation, mostly glycosylated xantho-cyaniding [17,18]. Various homologues of anthocyanin biosynthesis pathway genes are accumulated in the process of pigment accumulation in the rose petals of *R. chinensis* ‘Old Blush’ when buds redden rapidly [17], and the unigene of the Cy-3,5-diglucoside-catalyzing enzyme (*RhGT1*) [19] decreases in the fully flowering stage when the petal color becomes lighter. This is completely different from in petunia. The formation of white- or red-colored petunia flowers is determined by the enzymatic competition between *FLS* and *DFR*, showing a close relationship to the disequilibrium of *FLS* and *DFR* expression [20].

Loss of duplicate gene expression and function, *cis*- and *trans*-acting effects, RNA-mediated pathways, and regulatory networks, etc., might have resulted in commonly seen phenotypic variation in polyploid wild plants and domesticated crops [21]. Polyploidy results in an immediate elevation in gene dosage throughout the genome. With more gene copies, polyploids may have elevated (or altered) gene expression, which would exert potential influence on a lot of phenotypic traits [22]. Also, epigenetic-regulated gene silencing might also immediately affect the gene expression for polyploid plants [23]. For some dose-dependent genes, e.g., *ETH*, their expression has some differences between the diploid roses and the tetraploid roses [17]. As for the decaploid *R. praelucens*, there is nothing known for explaining its phenotypic diversity, especially the flower color variation. As far as gene expression is concerned, it is necessary to screen the key related genes and their intraspecific expression first for elucidating the color variation within this decaploid species, which will then also be helpful for us to understand the effect of the most popular ploidy level, tetraploidy, on the phenotypic variation in modern roses.

Quantitative real-time PCR (qRT-PCR) is the most prominent and extensively used technique to quantify the expression of candidate genes owing to its high sensitivity, sufficient reproducibility, and wide quantification range, as well as ease of use [24]. However, a lack of experimental standardization, including data normalization, directly influences the reproducibility and integrity of biological replications of qRT-PCR experiments [25]. Data normalization is often established by containing stably expressed reference genes to correct an assay for sample-to-sample variation. In addition, the most popular reference genes are the “housekeeping genes”, including actin (*ACT*), tubulin (*TUBA* and *TUBB*), eukaryotic translation elongation factor 1-α (*EEF1A*), ubiquitin (*UBQ*), and glyceraldehyde-3-phosphate dehydrogenase (*GAPDH*). However, more and more studies have indicated that many of these genes are inappropriate for qRT-PCR analysis, as their expression could probably be altered by developmental and environmental factors [26,27,28]. It is widely accepted that reference genes are specific to experimental situations and even the ploidy levels [29], and suitable reference genes have to be experimentally detected for each species and for each certain experiment [28,30,31].

In roses, *PP2A* and *UBC* were shown to be more stable and suitable than those traditional housekeeping genes as references for the study of stress [29]. Different genes were chosen as the references under different postharvest conditions for more accurate and widespread application of qRT-PCR analysis in rose flowers, e.g., *RhUBI1* for dehydration treatment and receptacles, *RhTUB2* for exogenous ethylene, *RhUBI6* for petals, etc. [32]. As for the expression of petal-color-related genes in roses, there is no special selection of reference genes and the traditional ones adopted for *Chrysanthemum* [33], such as *ACT* [17] and *GAPDH* [34], are often used.

In this work, based on the transcriptome data of *R. praelucens* samples, with the aim of studying petal-color-related genes and their expression, we selected seven candidate reference genes and investigated their expression stability. The results provide optimal reference genes for exploring the level of petal-color-related genes in *R. praelucens*, as well as in modern roses, and also make contributions to understand the molecular mechanisms of intraspecific flower color diversity in plants.

## 2. Materials and Methods

### 2.1. Plant Materials and Sampling

**Figure 1:**
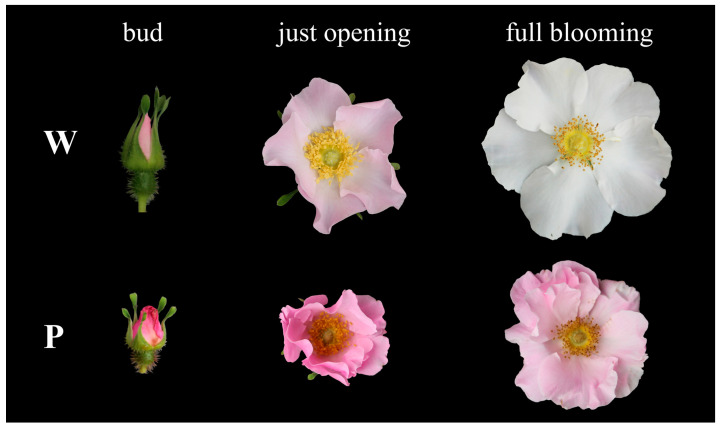
Phenotypic variation in *R. praelucens* and the samples used for RNA-seq and qRT-PCR validation. W: white phenotype; P: pink phenotype.

Flowers at three different floral development stages of *R. praelucens* with white and pink petals (Figure 1) were used for RNA-seq. All the sampled plants were naturally distributed in Xiaozhongdian town of Shangrila county in southwestern Yunnan Province, China. The environment and climate of their habitats were almost identical. The flowers at the same development stage removed of sepals and the bottom three-quarters of the hypanthium from one plant were mixed as one sample and immediately frozen in liquid nitrogen. Three plants with the same petal color at almost the same age and growth status were sampled for each replicate, and then a total of 18 samples were gathered and preserved at −80 °C within a refrigerator until further experiments were conducted.

### 2.2. Candidate Reference Genes, Petal-Color-Related Genes Selection, and Primer Design

Because color difference looks most significant and development is relatively stable at the full-blooming stage before senescence, the transcriptome dataset of the full-blooming stage was used for screening the candidate reference genes for color variation in line with the relative stability of the expression patterns of the genes (fragments per kb per million reads; FPKM) [35]. The seven commonly used candidate reference genes including tubulin α chain (*TUBA*), *GAPDH*, histoneH2B (*Histone2A*), *EEF1A*, 60S ribosomal protein (*RPL37*), eukaryotic translation initiation factor 1-α (*EIF1A*), and aquaporins (*AQP*) were selected to be potential reference genes for exploring the relative expression of petal-color-related genes in *R. praelucens* (Table 1). Additionally, according to the functional annotation of DEGs (differentially expressed genes) from the transcript analysis, six genes related to petal color variation at the full-blooming stage were assigned for validation of the most reliable reference genes. Expression of all of them was up-regulated in transcriptome libraries of pink-flowered *R. praelucens* compared with white ones (Table 2). Primers of the candidate reference genes and the petal-color-related genes were designed with the use of the Primer Premier 5.0 software [36], as presented in Table 3.

### 2.3. RNA Extraction

In line with the specifications of the manufacturer, the total RNA of each sample was extracted using the RNAQUEOUS KIT Ambion-1912 (Oebiotech, Shanghai, China). The extracted RNA concentration and quality (OD260/OD280 value) were evaluated using a NanoDrop 2000 spectrophotometer (Thermo Scientific, Waltham, MA, USA). Then, the integrity was confirmed with agarose gel electrophoresis (1%) stained with ethidium bromide.

### 2.4. Real-Time Quantitative RT-PCR

Quantification was carried out with a two-step reaction process including reverse transcription (RT) and PCR. Each RT reaction consisted of 0.5 μg RNA, 2 μL of 5× TransScript all-in-one SuperMix for qPCR, and 0.5 μL of gDNA Remover, in a total volume of 10 μL. The reactions were conducted within a GeneAmp^®^ PCR System 9700 (Applied Biosystems, Foster City, CA, USA). The reaction conditions were 15 min at 42 °C and 5 s at 85 °C. The 10 μL RT reaction mix was ten-time diluted with nuclease-free water, and then stored at −20 °C.

Real-time PCR was carried out with a LightCycler^®^ 480 II Real-time PCR Instrument (Roche, Basel, Switzerland) with 10 μL of PCR reaction mixture including 1 μL of cDNA, 5 μL of 2× PerfectStartTM Green qPCR SuperMix, 0.2 μL of forward primer, and 0.2 μL of reverse primer, as well as 3.6 μL of nuclease-free water. The primer sequences were synthesized by TsingKe Biotech, Beijing, China (Table 2). The reactions were subject to incubation within a 384-well optical plate (Roche, Basel, Switzerland) at 94 °C for 30 s, followed by 45 cycles of 94 °C for 5 s, 60 °C for 30 s. In addition, each sample was run in triplicate for performing the analysis. At the termination of the PCR cycles, a melting curve analysis was carried out, aiming to confirm the specific generation of the expected PCR product. Next, cycle threshold (Ct) values of each gene were automatically computed and then saved [37].

### 2.5. Analysis of Expression Stability

The expression stability of the candidate reference genes was assessed with three common software programs, geNorm version 3.4 [38], NormFinder version 20 [39], and BestKeeper version 1 [40], using the Ct values calculated from qRT-PCR data in accordance with the 2^−ΔΔCT^ method [41]. Firstly, geNorm was employed to compute the average expression stability (M value) of each candidate reference gene according to pairwise expression ratios and to identify the optimal number of reference genes by pairwise variation value (Vn/n+1, where n represents the reference gene number) [24,38]. Usually, a gene can be chosen as a reference gene when its M value is below 0.15. The lower the M value, the more stable the gene [37]. Secondly, NormFinder was applied to compute the stability value (SV) of the reference genes through analyzing the intra- and inter-group variation [39]. Those exhibiting a lower level of expression than the average SVs were thought to be the most stable candidate reference genes [37]. Bestkeeper, on the basis of the standard deviation value (SD) is usually further adopted for evaluating the expression stability of candidate reference genes [40]. The programs were run in line with their respective manuals’ instructions. The geometric mean method [42] was adopted for comprehensively ranking the expression stability of the candidate reference genes from the above software. Data analysis, statistics, and graphing were carried out by Microsoft Excel 2016 and Data Processing System [43]. We obtained statistical differences through an independent *t*-test at *p* < 0.05.

### 2.6. Validation of Reference Gene

Additionally, the reliability of the top reference gene at the comprehensive ranking list was validated by comparing the expression pattern of six petal-color-related target genes (Table 2) via qRT-PCR analysis normalized by the selected reference gene with that in the transcriptome analysis. In addition, the qRT-PCR method remained the same as mentioned above, with three biological replicates.

## 3. Results

### 3.1. Isolation of Candidate Reference Genes from Transcriptome Analysis

Based on the RNA-seq transcriptome data of *R. praelucens*, the FPKM value of the seven candidate reference genes are presented in Table 1. Except for the FPKMs of *TUBA* and *GAPDH*, which were a little bit lower than 60, those of the other genes were higher than 100 (Table S1).

### 3.2. Primer Specificity and Expression Profile Analyses of Candidate Reference Genes

**Figure 2:**
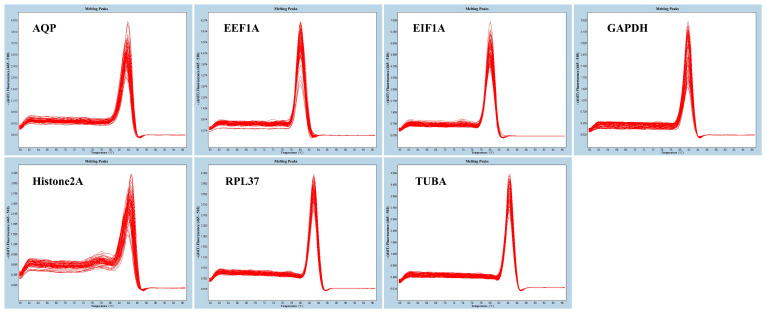
Melting curves of the seven candidate reference genes in *R. praelucens*.

The seven candidate reference genes were amplified through qRT-PCR experiments, aiming to confirm the specificity of their primers. Their melting curves all showed a distinctive single peak without a primer dimer (Figure 2). All amplicons revealed good repeatability, indicating that the primers had strong specificity, reliability, and accuracy. As a result, all of the specific primers were shown to be appropriate for performing further qRT-PCR detection.

### 3.3. Expression Profile Analysis of Candidate Reference Genes

**Figure 3:**
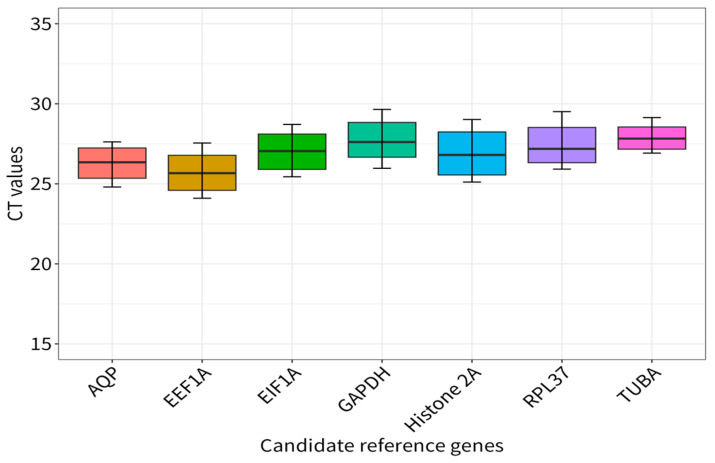
qRT-PCR Ct values for all candidate reference genes in six samples and 3 replicons each.

A Ct boxplot analysis (Figure 3) indicated that the Ct values of the seven candidate reference genes were within the range 25.69 ± 1.22 (*EEF1A*, *n* = 18) to 27.89 ± 0.79 (*TUBA*, *n* = 18), and that the average Ct values of *EIF1A*, *GAPDH*, *Histone2A*, *RPL37*, and *AQP* were 27.42 ± 1.24, 27.76 ± 1.27, 26.90 ± 1.46, 27.42 ± 1.24, and 26.28 ± 1.04, respectively. Among them, *TUBA* showed the least expression variation, from 26.92 to 29.14, with a coefficient value (CV) of 2.83%, followed by *AQP* from 24.80 to 27.62, with a CV of 3.96% (Table S2). The CVs of the others were 4.58% (*GAPDH*), 5.43% (*Histone2A*), 4.76% (*EEF1A*), 4.51% (*RPL37*), and 4.49% (*EIF1A*).

### 3.4. Stability Analysis of Candidate Reference Genes

**Figure 4:**
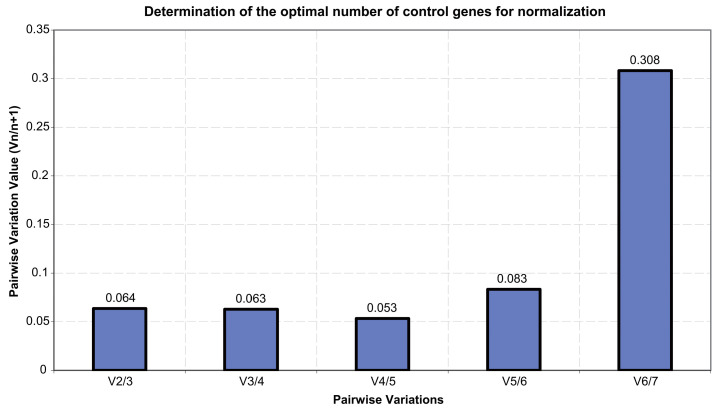
Pairwise variation analysis to identify the optimal number of reference genes in *R. praelucens.*

In accordance with the result of the pairwise variation value (Vn/n+1) between sequential normalization factors (Figure 4), because most of the Vn/n+1 values, e.g., V2/3, V3/4, V4/5, V5/6, are far less than 0.15, two reference genes might be required for the expression study of the target petal-color-related genes for *R. praelucens*.

The average gene expression stability values (M value; Table 4) evaluated by geNorm showed that *EEF1A* exhibited the lowest M value (0.576) and *EIF1A* had the second lowest one (0.585), while those of *RPL37*, *GAPDH*, *Histone2A*, and *TUBA* were 0.608, 0.656, 0.714, and 0.728, respectively, all lower than 1.5. Only the M value of *AQP* was much higher than 1.5. Thus, *EEF1A* and *EIF1A* can be regarded as the most stably expressed candidate reference genes. In addition, the other four might also be suitable.

The expression stability values (SVs) computed by NormFinder (Table 4) indicated that the average SV of the seven candidate reference genes was 0.375. So, *EEF1A*, *EIF1A*, *RPL37*, *GAPDH*, and *TUBA* were more stably expressed, while *Histone2A* and *AQP*, with SVs of 0.399 and 1.296, respectively, were unstable.

Among the seven candidate reference genes, only the standard deviation (SD) of the gene expression of *TUBA* and *AQP* calculated by BestKeeper was lower than 1.0. The SDs of *GAPDH* and *EEF1A* were 1.086 and 1.114, respectively, while those of *EIF1A*, *RPL37*, and *Histone2A* were even higher.

Despite geNorm and NormFinder generating almost the same results for the stable reference genes in *R. praelucens*, the result computed by BestKeeper was somewhat different, since the computation was based on different criteria. The result of the GM method adopted for integrating the results from the three methods confirmed that *EEF1A* was the most stable candidate reference gene with the lowest GM value of 1.587, followed by *EIF1A* and *TUBA*, both with a GM value of 2.884. They can be used as suitable and reliable reference genes for studying petal-color-related genes in *R. praelucens*, while *AQP* and *Histone2A* were not so stable.

### 3.5. Validation of Selected Reference Gene EEF1A

**Figure 5:**
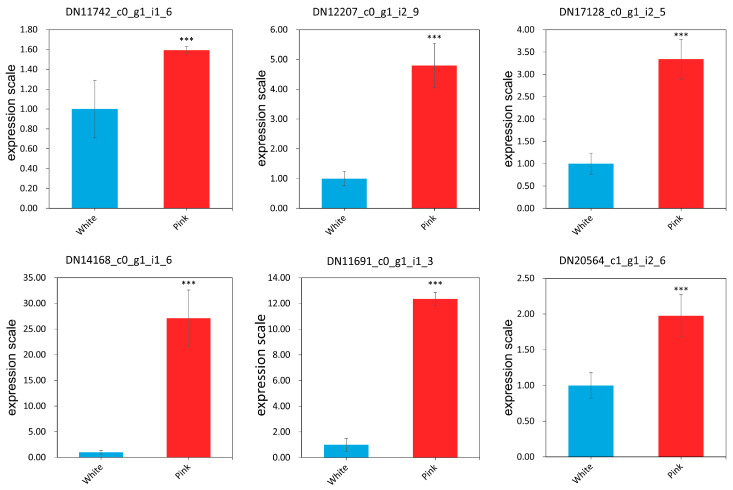
The qRT-PCR expression patterns of the petal-color-related candidate genes in the white- and pink-petaled individuals. Note: *** in the figures indicates statistical differences at *p* < 0.001. The *p*-values of DN11742_c0_g1_i1_6, DN12207_c0_g1_i2_9, DN17128_c0_g1_i2_5, DN14168_c0_g1_i1_6, DN11691_c0_g1_i1_3, and DN20564_c1_g1_i2_6 were 0.000018, 0.000000, 0.000000, 0.000000, 0.000000, and 0.000000, respectively.

To demonstrate the utility and stability of *EEF1A* as the reference gene in the study of petal-color-related genes in *R. praelucens*, the expression of the six selected target genes engaged in the petal color variation were evaluated. In addition, the expression pattern of these genes in the full-blooming flowers of white and pink individuals via qRT-PCR analysis, were normalized to that of the most suitable reference gene, *EEF1A*, according to the 2^−ΔΔCt^ method (Table S3). Based on the results, expression of the six petal-color-related genes was significantly up-regulated in the full-blooming flowers of pink-petal-colored individuals compared with their expression in those of the white ones (*p* < 0.01) (Figure 5), conforming to the result of transcriptome analysis (Table 2 and Table S4). The expression pattern of these possible target genes was consistent with their transcriptomic analysis of RNA-seq. Thus, *EEF1A* is an appropriate reference gene for the gene expression of the petal-color-related genes in *R. praelucens*.

## 4. Discussion

Flower color is one of the most prominent characteristics of roses [44]. With more gene copies, polyploids may have changed gene expression, and thus, affected many phenotypic traits [22]. The decaploid *R. praelucens* has rich intra-species diversity in flower color, from white to different degrees of pink [6,14]. The mechanism underlying this, especially the expression of related genes, needs to be deeply investigated. The expression of the dose-dependent genes has some differences between the diploid roses and the tetraploid ones [17,29]. For the expression analysis of flavonoid/anthocyanin synthesis pathway-related genes, different reference genes have been used, e.g., *AQP* in *Iris hollandica* [45,46], *ACT* in *Muscari botryoides* [47] and in *R. chinensis* (‘Old Blush’) [17], 18S in hybrid *Dendrobium* [48], and *GAPDH* in hybrid roses [34], but none of them were specifically selected and validated.

Herein, we screened seven candidate reference genes in *R. praelucens*, including *EEF1A*, *EIF1A*, *TUBA*, *GAPDH*, *AQP*, *Histone2A*, and *RPL37*, and revealed that *EEF1A* was the most appropriate reference gene for the expression of petal-color-regulated genes. The expression pattern of six petal-color-regulated genes, determined via qRT-PCR analysis, using *EEF1A* as reference gene for normalization, were consistent with the transcriptome sequencing result, confirming the reliability and suitability of applying it as the reference gene in *R. praelucens*. The selected new reference gene in this study, *EEF1A*, was different from those that had previously been screened and validated for the petal-color-related gene expression of either Asiatic hybrid lilies, i.e., *TUB*, [49], or *Anemone obtusiloba*, i.e., *UBQ* [50], which reconfirmed that suitable reference genes had to be detected for each species and for each experiment [28,30,31].

Most flower colors in roses, especially the pink and red, are determined by chemicals such as flavonoids and anthocyanins [44,51,52,53,54]. The differences in petal color, brightness, and saturation of modern roses are mainly due to the change in expression quantity of genes such as *CHS*, *DFR*, and *ANS*, etc. [34]. The *FLS* gene in *R. rugosa* ‘Hunchun’ is closely related to that in *R. multiflora* [55]. Different homologues of anthocyanin biosynthesis pathway genes are related to the petal color variation in *R. chinensis* ‘Old Blush’ [17]. As far as the key transcription factors are concerned, *MYB*, *bHLH*, and *WD40* are the three most possible transcription factors regulating the expression of key enzyme genes in plants [56,57,58]. Among them, *MYB* transcription factors, such as *MYB1* [59,60] and *R2R3-MYB* [61,62] have been proved to be key transcriptors in rose anthocyanin biosynthesis. Among the six candidate petal-color-related genes, DN11691_c0_g1_i1_3, DN14168_c0_g1_i1_6, and DN12207_c0_g1_i2_9 were the most significantly up-regulated genes in the pink-petaled individuals. Therefore, these genes may indicate key candidate genes or transcriptors for deciphering the petal color variation in *R. praelucens*. As a result, functional characterization of these genes is needed in order to show their roles and clarify petal color molecular mechanisms in *R. praelucens*. The discovery of reference genes may speed up the molecular dissection of petal-color-variation mechanisms in *R. praelucens*.

## 5. Conclusions

To conclude, *R. praelucens* Byhouwer is an endemic and critically endangered decaploid wild rose species. It has rich intraspecific flower color variation from white to different degrees of pink. With the purpose of selecting reference gene for further study on the expression of petal-color-related genes, seven candidate reference genes, screened from a transcriptome dataset for qRT-PCR normalization of expression of petal-color-related target genes in *R. praelucens*, were investigated. *EEF1A* was shown to be the most stable reference gene. Its reliability was validated via qRT-PCR analysis of the expression pattern of six petal-color-related DEGs, which were identical to the RNA-seq results. DN11691_c0_g1_i1_3, DN14168_c0_g1_i1_6, and DN12207_c0_g1_i2_9 were the most obviously up-regulated genes in the pink flowers. They might be the vital target genes for the intraspecific petal color variation in *R. praelucens*.

## Figures and Tables

**Table 1:** The details of candidate reference genes screened from transcriptome libraries of *R. praelucens.*

Genes	FPKM	Nr Annotation
TUBA	48.88 ± 6.10	tubulin α chain
GAPDH	44.27 ± 16.74	glyeraldehyde 3-phosphate dehydrogenase
Histone2A	104.97 ± 34.30	histone H2B
RPL37	164.71 ± 37.83	60S ribosomal protein L37-3-like
EEF1A	113.08 ± 60.23	elongation factor 1-α
EIF1A	157.89 ± 39.51	eukaryotic translation initiation factor 1A
AQP	276.73 ± 197.22	aquaporin PIP2-1-like

**Table 2:** FPKM of the petal-color-related genes in the full opening flowers of different *R. praelucens.*

Gene ID	White	Pink	*p*-Value #	Gene Expression
DN11742_c0_g1_i1_6	0.981 ± 0.416	24.618 ± 5.141	0.0014 **	Up-regulated
DN12207_c0_g1_i2_9	1.591 ± 0.096	46.562 ± 8.123	0.00067 ***	Up-regulated
DN17128_c0_g1_i2_5	3.281 ± 0.398	37.732 ± 1.513	2.825 × 10^−6^ ***	Up-regulated
DN14168_c0_g1_i1_6	0.185 ± 0.095	7.524 ± 0.312	2.582 × 10^−6^ ***	Up-regulated
DN11691_c0_g1_i1_3	0.282 ± 0.164	18.862 ± 0.395	1.875 × 10^−7^ ***	Up-regulated
DN20564_c1_g1_i2_6	0.344 ± 0.300	2.380 ± 0.452	0.0029 **	Up-regulated

#: ** and *** suggest statistical differences at *p* < 0.01 and 0.001, respectively.

**Table 3:** Primer sequences for the qRT-PCR expression analysis of candidate reference genes and petal-color-related genes of *R. praelucens.*

Type	Gene	Forward Primer (5->3)	Reverse Primer (5->3)	Length (bp)	Tm (°C)
Candidate reference genes	GAPDH	GATGAGGATGTTGTGTCAACC	GCTTGACAAACTTGTCGTTC	97	60
	TUBA	CAGCCTGATGGCCAAATG	CAACAAAGATTGCGCGAG	115	60
	Histone 2A	TTACTTCCCGGGAGATCCAG	CTTAGTCCCCTCAGAAACGG	83	60
	EEF1A	ATCCTCACCAAGATTGACAG	TTGGTGGGAAGCATCTTAAC	101	60
	RPL37	TGAGGTACCTTCGCCATGTG	TTCCCTTGCTCCTTGGCTTA	81	60
	EIF1A	CTTCCAGAGAATACACGTCTTA	CTCGATGTAGTCATCAGCAC	84	60
	AQP	TCTCAGCCAAGGACTACC	AGCACAGTGATGTAGAGAAAC	133	60
Petal-color-related genes	DN11742_c0_g1_i1_6	CTAAAGAGGTTGAGAGCGCTA	TACAAAGGATAAGACCTGCAC	91	60
	DN12207_c0_g1_i2_9	CCAAGGAGAAGATCATCCTCA	GGATATGCTCGGAGAAAGTC	93	60
	DN17128_c0_g1_i2_5	TTGACCAAGGCATGTGTGGA	TAATTGTACCCGGATCTGTGT	115	60
	DN14168_c0_g1_i1_6	GTGATGACTTGGTGTACCTT	GTTTGGGACGAGAATGGTGA	119	60
	DN11691_c0_g1_i1_3	TGCATGGACTAAAGAGGAAGA	AAAGGAACCTGATGCCATT	81	60
	DN20564_c1_g1_i2_6	CAAGCTACCGATAGCCACA	CCTTATTACATCCGGGAACCA	100	60

**Table 4:** Stability ranking of candidate reference genes in *R. praelucens* using geNorm, NormFinder, BestKeeper software and the geometric mean (GM) method.

Gene	geNorm		NormFinder		BestKeeper		Geometric Mean	
	M Value	Ranking	SV	Ranking	SD	Ranking	Geomean	Comprehensive Ranking
*EEF1A*	0.576	1	0.109	1	1.114	4	1.587	1
*EIF1A*	0.585	2	0.122	2	1.127	6	2.884	2
*RPL37*	0.608	3	0.144	3	1.120	5	3.557	3
*GAPDH*	0.656	4	0.205	5	1.086	3	3.915	4
*Histone2A*	0.714	5	0.399	6	1.353	7	5.944	6
*TUBA*	0.728	6	0.151	4	0.730	1	2.884	7
*AQP*	2.162	8	1.494	7	0.968	2	4.610	8

## Data Availability

In this work, all the datasets explored or generated are contained in the current study as well as its Supplementary File.

## Supplementary Materials

The following supporting information can be downloaded at: https://www.mdpi.com/article/10.3390/genes15030277/s1, Table S1: List of the seven candidate reference genes with their FPKM expression values; Table S2: Summary of the qRT-PCR Ct values of seven candidate reference genes; Table S3: qRT-PCR Ct values of the six target petal-color-related genes; Table S4: List of the six petal-color-related genes with their FPKM expression values.

## References

[1] Ku T.C., Robertson K.R., Wu Z.Y., Raven P.H. (2003). Rosa (Rosaceae). Flora of China.

[2] Qin H.N., Yang Y., Dong S.Y., He Q., Jia Y., Zhang L.N., Yu S.X., Liu H.Y., Liu B., Yan Y.H. (2017). Threatened species list of China’s higher plants. Biodivers. Sci..

[3] Li X.X., Zhou Z.K. (2015). Endemic wild ornamental plants from North Western Yunnan. HortScience.

[4] Deng J.Q., Jian H.Y., Li S.B., Wang Q.G., Guo Y.L., Zhang H. (2013). Cold tolerance of several wild Rosa resources endemic to Yunnan. Southwest. Chin. J. Agric. Sci..

[5] Fan Y.L., Chen Y.C., Jian H.Y., Yan H.J., Zhang T., Li S.F., Qiu X.Q. (2021). Screening of *Rosa germplasm* resources with resistance to aphids. J. Yunnan Univ. (Nat. Sci. Ed.).

[6] Jian H.Y., Li S.F., Guo J.L., Li S.B., Wang Q.G., Yan H.J., Qiu X.Q., Zhang Y.H., Cai Z.Q., Volis S. (2018). High genetic diversity and differentiation of an extremely narrowly distributed and critically endangered decaploid rose (*Rosa praelucens*): Implications for its conservation. Conserv. Genet..

[7] Wu X.Y., Chen M., Wang Q.G., Zhou N.N., Zhang T., Yan H.J., Qiu X.Q., Li S.B., Zhang H., Jian H.Y. (2014). Comparative study on the breeding systems of *Rosa praelucens* and *Rosa soulieana*. Acta Hortic. Sin..

[8] Pan L.J., Guan W.L., LI Y.H. (2019). Seed dormancy mechanism and its ecological significance of endangered species *Rosa praelucens*. Subtrop. Plant Sci..

[9] Wang K.J., Zhang T., Wang Q.G., Yan H.J., Qiu X.Q., Li S.B., Zhang H., Tang K.X., Jian H.Y. (2018). The phylogenetic position and hybrid origination of *Rosa praelucens* Byhouwer. J. Plant Genet. Resour..

[10] Fang Q., Tian M., Zhang T., Wang Q.G., Yan H.J., Qiu X.Q., Zhou N.N., Zhang H., Jian H.Y., Tang K.X. (2020). Karyotype analysis of *Rosa praelucens* and its closely related congeneric species based on FISH. Acta Hortic. Sin..

[11] Jian H.Y., Zhang H., Tang K.X., Li S.F., Wang Q.G., Zhang T., Qiu X.Q., Yan H.J. (2010). Decaploidy in *Rosa praelucens* Byhouwer (Rosaceae) endemic to Zhongdian Plateau, Yunnan, China. Caryologia.

[12] Pan L.J., Guan W.L., LI Y.H. (2018). Population structure and spatial distribution pattern of endangered species *Rosa praelucens*. Subtrop. Plant Sci..

[13] Guan W.L., Li S.F., Song J., Pan L.J., Niu H.B. (2012). Study on geographic distribution of *Rosa praelucens* endemic to Yunnan. J. W. Chin. For. Sci..

[14] Li S.F., Li C.J., Jian H.Y., Li S.B., Xiong J., Li J.K., Tang K.X. (2013). Studies on phenotypic diversity of vulnerable *Rosa praelucens* endemic to Shangrila, Yunnan. Acta Hortic. Sin..

[15] Smulders M.J.M., Arens P., Bourke P.M., Debener T., Linde M., De RieK J., Leus L., Ruttink T., Baudino S., Saint-Oyant H.L. (2019). In the name of the rose: A roadmap for rose research in the genome era. Hortic. Res..

[16] Okitsu N., Noda N., Chandler S., Tanaka Y., Van Huylenbroeck J. (2018). Flower color and its engineering by genetic modification. Ornamental Crops, Handbook of Plant Breeding.

[17] Han Y., Wan H.H., Cheng T.R., Wang J., Yang W.R., Pan H.T., Zhang Q.X. (2017). Comparative RNA-seq analysis of transcriptome dynamics during petal development in *Rosa chinensis*. Sci. Rep..

[18] Jay M., Biolley J.P., Fiasson J.L., Fiasson K., Gonnet J.F., Grossi C., Raymond O., Viricel M.R., Roberts A.V., Debener T., Gudin S. (2003). Anthocyanins and other flavonoid pigments. Encyclopedia of Rose Science.

[19] Ogata J., Kanno Y., Itoh Y., Tsugawa H., Suzuki M. (2005). Anthocyanin biosynthesis in roses. Nature.

[20] Luo P., Ning G.G., Wang Z., Shen Y.X., Jin H.N., Li P.H., Huang S.S., Zhao J., Bao M.Z. (2015). Disequilibrium of flavonol synthase and dihydroflavonol-4-reductase expression associated tightly to white vs. red color flower formation in plants. Front. Plant Sci..

[21] Chen Z.J. (2007). Genetic and epigenetic mechanisms for gene expression and phenotypic variation in plant polyploids. Annu. Rev. Plant Biol..

[22] Ramsey J., Schemske D.W. (2002). Neopolyploidy in flowering plants. Annu. Rev. Ecol. Syst..

[23] Wendel J.F. (2000). Genome evolution in polyploids. Plant Mol. Biol..

[24] Derveaux S., Vandesompele J., Hellemans J. (2010). How to do successful gene expression analysis using real-time PCR. Methods.

[25] Bustin S.A., Benes V., Garson J.A., Hellemans J., Huggett J., Kubista M., Mueller R., Nolan T., Pfaffl M.W., Shipley G.L. (2009). The MIQE guidelines: Minimum information for publication of quantitative realtime PCR experiments. Clin. Chem..

[26] Volkov R.A., Panchuk I.I., Schoffl F. (2003). Heat-stress-dependency and developmental modulation of gene expression: The potential of housekeeping genes as internal standards in mRNA expression profiling using real-time RT-PCR. J. Exp. Bot..

[27] Czechowski T., Stitt M., Altmann T., Udvardi M.K., Scheible W.R. (2005). Genome-wide identification and testing of superior reference genes for transcript normalization in arabidopsis. Plant Physiol..

[28] Wan Y.L., Hong A.Y., Zhang Y.X., Liu Y. (2019). Selection and validation of reference genes of *Paeonia lactiflora* in growth development and light stress. Physiol. Mol. Biol. Plants.

[29] Klie M., Debener T. (2011). Identification of superior reference genes for data normalisation of expression studies via quantitative PCR in hybrid roses (*Rosa hybrid*). BMC Res. Notes.

[30] Hruz T., Wyss M., Docquier M., Pfaffl M.W., Masanetz S., Borghi L., Verbrugghe P., Kalaydjieva L., Bleuler S., Laule O. (2011). RefGenes: Identification of reliable and condition specific reference genes for RT-qPCR data normalization. BMC Genom..

[31] Yi S.J., Qian Y.Q., Han L., Sun Z.Y., Fan C.M., Liu J.X., Ju G.S. (2012). Selection of reliable reference genes for gene expression studies in *Rhododendron micranthum* Turcz. Sci. Hortic..

[32] Meng Y.L., Li N., Tian J., Gao J.P., Zhang C.Q. (2013). Identification and validation of reference genes for gene expression studies in postharvest rose flower (*Rosa hybrida*). Sci. Hortic..

[33] Yan J.F. (2014). Studies on Metabolism and Variation Mechanism of Chimeric pigment in Chrysanthemum Flower Color. Master’s Thesis.

[34] Li Y.P., Zhang Y.J., Zhu Z.Q., Zhi J.W., Liu M.X., Zhang J.W., Guo W.J., Sun Y., Kong J.J., Sun J.X. (2022). Study on the Quantitative Analysis of 11 Key Genes Expression of Flower Color in Different Color System of Rosa. Mol. Plant Breed..

[35] Mortazavi A., Williams B.A., McCue K., Schaeffer L., Wold B. (2008). Mapping and quantifying mammalian transcriptomes by RNA-Seq. Nat. Methods.

[36] Lalitha S. (2000). Primer premier 5. Biotechnol. Softw. Internet Rep..

[37] Chen C.B., Wu J.Y., Hua Q.Z., Tel-Zur N., Xie F.F., Zhang Z.K., Chen J.Y., Zhang R., Hu G.B., Zhao J.T. (2019). Identification of reliable reference genes for quantitative real-time PCR normalization in pitaya. Plant Meth..

[38] van Vandesompele J., Preter K., Pattyn F., Poppe B., Roy N., Paepe A., Speleman F. (2002). Accurate normalization of real-time quantitative RT-PCR data by geometric averaging of multiple internal control genes. Genome Biol..

[39] Andersen C.L., Jensen J.L., Ørntoft T.F. (2004). Normalization of real-time quantitative reverse transcription-PCR data: A model-based variance estimation approach to identify genes suited for normalization, applied to bladder and colon cancer data sets. Cancer Res..

[40] Pfaffl M.W., Tichopad A., Prgomet C., Neuvians T.P. (2004). Determination of stable housekeeping genes, differentially regulated target genes and sample integrity: BestKeeper–excel-based tool using pair-wise correlations. Biotechnol. Lett..

[41] Chen D., Pan X., Xiao P., Farwell M.A., Zhang B. (2011). Evaluation and identification of reliable reference genes for pharmacogenomics, toxicogenomics, and small RNA expression analysis. J. Cell Physiol..

[42] Livak K.J., Schmittgen T.D. (2001). Analysis of relative gene expression data using Real-Time Quantitative PCR and the 2_−∆∆CT_ Method. Methods.

[43] Tang Q.Y., Zhang C.X. (2013). Data processing system (DPS) software with experimental design, statistical analysis and data mining developed for use in entomological research. Insect Sci..

[44] Wan H.H. (2018). Identification and QTL Analysis of Flavonoids and Carotenoids in Rose Petals. Ph.D. Thesis.

[45] Yoshihara N., Imayama T., Fukuchi-Mizutani M., Okuhara H., Tanaka Y., Ino I., Yabuya T. (2005). cDNA cloning and characterization of UDP-glucose: Anthocyanidin 3-O-glucosyltransferase in *Iris hollandica*. Plant Sci..

[46] Yoshihara N., Imayama T., Matsuo Y., Fukuchi-Mizutani M., Tanaka Y., Ino I., Yabuya T. (2006). Characterization of cDNA clones encoding anthocyanin 3-p-coumaroyltransferase from *Iris hollandica*. Plant Sci..

[47] Lou Q., Liu Y.L., Qi Y.Y., Jiao S.Z., Tian F.F., Jiang L., Wang Y.J. (2014). Transcriptome sequencing and metabolite analysis reveals the role of delphinidin metabolism in flower color in grape hyacinth. J. Exp. Bot..

[48] Kriangphan N., Vuttipongchaikij S., Kittiwongwattana C., Suttangkakul A., Pinmanee P., Sakulsathaporn A., Suwimon R., Suputtitada S., Chanvivattana Y., Apisitwanich S. (2015). Effects of sequence and expression of eight anthocyanin biosynthesis genes on floral coloration in four *Dendrobium* hybrids. Hortic. J..

[49] Xu L.F., Xu H., Cao Y.W., Yang P.P., Feng Y.Y., Tang Y.C., Yuan S.X., Ming J. (2017). Validation of reference genes for quantitative real-time PCR during bicolor tepal development in asiatic hybrid lilies (*Lilium* spp.). Front. Plant Sci..

[50] Ma L.L., Duan Q., Cui G.F., Du W.W., Jia W.J., Wang X.N., Wang J.H., Chen F.D. (2021). Selection and validation of reference genes for qRT-PCR analysis of the correlated genes in flower pigments biosynthesis pathway of *Anemone obtusiloba*. Acta Hortic. Sin..

[51] Biolley J.P., Jay M. (1993). Anthocyanins in modern roses: Chemical and colorimetric features in relation to the color range. J. Exp. Bot..

[52] Yuki M., Yokoi M., Ueda Y., Saito N. (2000). Anthocyanins in flowers of genus Rosa, sections *Cinnamomeae* (=*Rosa*), *Chinenses*, *Gallicanae* and some modern garden roses. Biochem. Syst. Ecol..

[53] Sarangowa O., Kanazawa T., Nishizawa M., Yamagishi T. (2014). Flavonol glycosides in the petal of Rosa species as chemotaxonomic markers. Phytochemistry.

[54] Cai Y.Z., Xing J., Sun M., Corke H. (2005). Phenolic antioxidants (hydrolyzable tannins, flavonols, and anthocyanins) identified by LC-ESI-MS and MALDI-QIT-TOF MS from *Rosa chinensis* flowers. J. Agric. Food Chem..

[55] Li Z.J., Zhang P.Y., Jin J.F., Zhang L.Y., Xu Z.D. (2018). Cloning and bioinformatics analysis of flavonol synthase gene in *Rosa rugosa*. Shandong Forest. Sci. Technol..

[56] Meng J.X., Gao Y., Han M.L., Liu P.Y., Yang C., Shen T., Li H.H. (2020). In Vitro anthocyanin induction and metabolite analysis in *Malus spectabilis* leaves under low nitrogen conditions. Hortic. Plant J..

[57] Fu Z.Z., Shang H.Q., Jiang H., Gao J., Dong X.Y., Wang H.J., Li Y.M., Wang L.M., Zhang J., Shu Q.Y. (2020). Systematic identification of the light-quality responding anthocyanin synthesis-related transcripts in petunia petals. Hortic. Plant J..

[58] Ramsay N.A., Glover B.J. (2005). MYB-bHLH-WD40 protein complex and the evolution of cellular diversity. Trends Plant Sci..

[59] Xie J.R., Xiong Y.H., Cheng Z.Q., Huang X.Q. (2008). Full length cloning and expression analysis of MYB gene cDNA of *Rosa chinensis*. Chin. Agric. Sci..

[60] He G.R., Zhang R., Jiang S.H., Wang H.H., Ming F. (2023). The MYB transcription factor RcMYB1 plays a central role in rose anthocyanin biosynthesis. Hortic. Res..

[61] Zhao J., Liu R., Yang F., Li X., Liu H.S., Yan Q., Xiao Y.H. (2015). Cloning and expression analyses of R 2R3-MYB genes related to anthocyanin biosynthesis in Rose. Chin. Agric. Sci..

[62] Li M.F., Yang Y., Wang H., Fan Y.W., Sun P., Jin W.M. (2022). Analysis the function of R2R3 MYB transcription factor RhMYB113 on regulating anthocyanin synthesis in *Rosa hybrida*. Acta Hortic. Sin..

